# Comparative Study on the Crystallography of Isothermal and Athermal Precipitations in HCP–BCC System

**DOI:** 10.3390/ma15217484

**Published:** 2022-10-25

**Authors:** Hongwei Liu

**Affiliations:** Sydney Microscopy & Microanalysis, The University of Sydney, City Road, Sydney, NSW 2006, Australia; hongwei.liu@sydney.edu.au

**Keywords:** isothermal and athermal precipitation, crystallography of phase transformations, habit plane, growth direction, orientation relationship

## Abstract

**Simple Summary:**

A comparison study on the crystallography of isothermal and athermal phase transformations between hexagonal close-packed structure and body-centred cubic structure was systematically investigated in a full range of a lattice parameter ratio.

**Abstract:**

Due to the diversity of the lattice parameter ratio *c*/*a* of hexagonal structure and precipitation mechanism, a systematic overview of this transformation has not been fully established, which draws back the attempt to control crystallographic features of the precipitates and microstructures of applied metals and alloys. Here, a comparative investigation to the crystallography of isothermal and athermal precipitations occurring in the HCP–BCC system was demonstrated in a full range of the lattice parameter ratio by using an invariant deformation element (IDE) model. It was then proposed that a precipitation in the HCP–BCC system could be either of the isothermal type if the observed habit plane Miller index falls into a zone axis of BCC <11w>(w ≠ 0) or HCP $\left[2\bar{11}0\right]$, or the athermal type when it is found to locate in a zone axis of BCC <11w](w = 0) or HCP [0001]. The crystallographic investigation on the precipitations in the HCP–BCC system in a full range of the lattice parameter may be a practical guide for computing material science when building a crystallographic interface model under an optimised orientation relationship, which is necessary to minimise the transformation system energy.

## 1. Introduction

Engineering structural materials are mainly metals and alloys with dominant structures of body-centred cubic (BCC), face-centred cubic (FCC), and hexagonal close-packed (HCP) lattices. Solid-state phase transformations often occur in metallic engineering materials with close-packed or nearly close-packed structures. Diffusional phase transformations in metals and alloys are of great research interest due to the important role of precipitation in enhancing the mechanical properties of metals and alloys. The HCP–BCC system is one of the most extensively studied diffusional phase transformations in terms of crystallographic features covering orientation relationship (OR), growth direction (GD), and habit plane (HP). Such transformation has been frequently observed in quite a few HCP–BCC transformation systems, such as *α*-Mg⟶*γ*-Mg_17_Al_12_ in the Mg–9 wt%Al–1 wt%Zn alloy AZ91 [1,2], *β*-Ti⟶ *α*-Ti in the Ti–6.62 at.% Cr alloy [3,4], *α*-Zr ⟶ *β*-Nb in the Zr–2.5 wt%Nb alloy [5,6], and *α*-Fe⟶Mo_2_C in the Fe–Mo–C alloy [7,8]. The crystallographic features of phase transformations in HCP–BCC systems were reported in detail in the above literature. Most of the observed OR is a typical Burgers OR, as reported by Burgers in 1934 [9]. Most of these works are based on the Pitch–Schrader orientation relationship, which defines a lattice correspondence between the HCP and BCC structure. However, successful interpretation of the crystallographic features of HCP–BCC transformations is case-sensitive. To the current author’s best knowledge, there is no systematic crystallographic investigation to a full range of a lattice parameter ratio (LPR) of HCP–BCC diffusional phase transformations in the literature. This is mainly because multiple variants of the LPR are in need of consideration (variants *γ_1_* = *a_b_*/*a_h_* and *γ_2_* = *c_h_*/*a_h_*, ‘*_b_*’ and ‘*_h_*’ denote ‘BCC’ and ‘HCP’, respectively). As a comparison, the LPR for FCC–BCC phase transformations has only one variant, i.e., *γ* = *a_f_*/*a_b_*, where ‘*_f_*’ denotes ‘FCC’.

The study on this system is often hindered by a few more practical issues. The crystallographic calculation of hexagonal lattice is not as easy as that of a cubic structure. The HCP–BCC diffusional phase transformation system is not as many as those for the FCC–BCC system. Another difficulty lies in the diversity of the precipitation mechanism between isothermal precipitation (*γ*-Mg_17_Al_12_/*α*-Mg) and athermal precipitation (ω-Ti/β-Ti, ω-Zr/α-Zr due to high hydrostatic pressure [10]). Recall that athermal means no thermal activation or deformation–activation and may happen in a large or even all temperature range; consequently, it does not depend on time with no need to wait for sufficient statistical fluctuations. On the contrary, isothermal precipitation is temperature-related and in need of thermal activation. For instance, the athermal ω phase in zirconium alloy is associated with rapid cooling from the β phase (BCC) region to room temperature, whereas the isothermal ω phase (HCP) is associated with isothermal aging within the approximate temperature range of 303–577 K [10,11]. The addition of the third element Zr was found to suppress isothermal ω formation in the β-Ti–Nb alloy [12]. One more example is the ω-Ti phase formation in the β-Ti alloy. It was found that the formation of isothermal ω in the Ti–Nb alloy could be suppressed by the addition of the third element Zr; meanwhile, it could also be promoted by deformation strain in the Ti–Cr alloy containing the athermal ω phase [13]. It is consequently necessary to differentiate the nature of the ω phase from an isothermal one to an athermal one by comparative study.

To attempt a comparative study on thermal and athermal precipitation of the HCP–BCC system, this work aimed to overcome the above difficulties by utilising the invariant deformation element (IDE) model [14], which has been successfully applied to HCP–BCC diffusional systems (*γ*-Mg_17_Al_12_/*α*-Mg [15]). This study may provide an alternative solution to a full-range distribution of the crystallographic features of complicated diffusional phase transformations and a guide on microstructure designation and controlling of engineering metallic materials. This study is also expected to be a helpful guide to identify an observed precipitation in the HCP–BCC system between the isothermal and athermal type by checking the habit plane Miller index as proposed at the end of this work.

## 2. Crystallographic Investigation to HCP–BCC Diffusional Phase Transformation Systems

According to the IDE model for diffusional phase transformations [14], precipitation can be regarded as diffusional phase transformation under the constraint that (1) the interfacial dislocation vector **P_1_** is non-inclined, though the module may change, and (2) there must be an invariant normal **Q_1_** perpendicular to the non-inclined vector **P_1_**.

(1) $$P_{1}\cdot Q_{1}=0$$

It keeps invariant during the transformation.

(2) $$\left|Q_{1}\right|=\left|Q_{2}\right|$$

**Figure 1:**
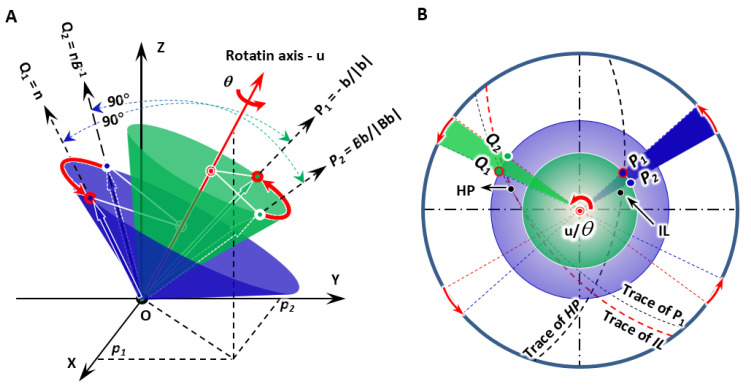
Scheme of IDE model showing the constraint of one-step rotation in diffusional phase transformation. (**A**) 3D model giving non-inclined line **P_1_**, invariant normal **Q_1_,** and the deformed lines **P_2_** and **Q_2_**. They separately attach on the surface of blue and green cones. (**B**) Stereographic projection presenting geometric configuration of all vectors, predicted growth direction (IL), habit plane (HP), and their traces.

As shown in Figure 1A, given that both non-inclined vector **P_1_** and invariant normal **Q_1_** are defined, it is easy to deduce a rotation matrix ***R***(**u**/*θ*) (Here, **u** is the unit rotation axis and *θ* is the rotation angle.) obtained by resolving Euler’s equation dealing with rigid-body rotation [16].

(3) $$R=u\cdot tan\left(\frac{\theta }{2}\right)=\frac{\left(P_{2}-P_{1}\right)\times \left(Q_{2}-Q_{1}\right)}{\left(P_{2}+P_{1}\right)\cdot \left(Q_{2}-Q_{1}\right)}$$

where the parameter ***R*** is the rotation matrix; **u** refers to a unit length vector parallel to the rotation axis; *θ* is the rotation angle; **P_1_** and **P_2_** are the real-space vector pair before and after rotation, respectively; and **Q_1_** and **Q_2_** are the reciprocal-space vector pair before and after rotation, respectively.

The rotation matrix ***R*** around a unitary axis [*p_1_*, *p_2_*, *p_3_*] about an angle *θ* is expressed as follows:

(4) $$R=\left(r_{ij}\right)=\left(\begin{array}{ccc} P_{1}^{2}\left(1-a\right)+a & P_{1}P_{2}\left(1-a\right)-P_{3}b & P_{1}P_{3}\left(1-a\right)\mp P_{2}b\\ P_{2}P_{1}\left(1-a\right)+P_{3}b & P_{2}^{2}\left(1-a\right)+a & P_{2}P_{3}\left(1-a\right)-P_{1}b\\ P_{3}P_{1}\left(1-a\right)-P_{2}b & P_{3}P_{2}\left(1-a\right)+P_{1}b & P_{3}^{2}\left(1-a\right)+a \end{array}\right),\;i,j\in 1,\;2,\;3$$

where the parameter *θ* is the rotation angle; *p_1_*, *p_2_*, and *p_3_* are the cosine components of a unitary vector parallel to the rotation axis; and *a* = cos*θ* and *b = sinθ*.

The one-step rotation operation defines the final OR between the product and matrix. The total transformation strain matrix ***A*** can now be composed as a matrix multiple of rotation matrix ***A*** and Bain strain ***B***:

(5) $$A=RB=\left(a_{ij}\right)=,\;i,j\in 1,\;2,\;3$$

All the three eigenvalues, eigenvectors, and eigenplanes can be achieved by linear algebra processing. Figure 1B shows the predicted invariant line (IL), i.e., GD and HP. It is immediately observed that the non-inclined line **P_1_** and the growth direction lie on the trace of the habit plane, whereas the invariant normal departs from the trace of the invariant line.

The invariant line IL is the cross-product of any two nonparallel rows of the matrix ***A*:**

(6) $$\mathrm{IL}=\left(\begin{array}{c} a_{11}\\ a_{12}\\ a_{13} \end{array}\right)\times \left(\begin{array}{c} a_{21}\\ a_{22}\\ a_{23} \end{array}\right)$$

The invariant normal **n** is the cross-product of any two nonparallel columns of the matrix ***A*:**

(7) $$n=\left(\begin{array}{c} a_{11}\\ a_{21}\\ a_{31} \end{array}\right)\times \left(\begin{array}{c} a_{12}\\ a_{22}\\ a_{32} \end{array}\right)$$

The habit plane HP is the cross-product of the invariant line and non-inclined line **P_1_**:

(8) $$\mathrm{HP}=IL\times P_{1}$$

To find the non-inclined line and non-inclined plane normal in an HCP–BCC precipitation system, it must be clear that there are two different precipitation mechanisms for this type of a precipitation system, i.e., isothermal and athermal precipitation [17]. The former is fully controlled by interfacial diffusion, and, consequently, it is enough to find a Burgers vector from the matrix and define it as a non-inclined line. Examples are the BCC-Nb/HCP-Zr and BCC-Mg_17_Al_12_/HCP-Mg systems. On the other hand, athermal precipitation involves a shear on the close-packed plane along the close-packed direction of the matrix with a condition that the shear plane does not change orientation. It is apparent that this is different from displacive phase transformation (e.g., martensitic transformation) that acquires an invariant line in a shear plane and an invariant normal in a shear direction. Practically, isothermal precipitation is identical to diffusional phase transformation, whereas athermal precipitation is a mixture of diffusion and displacive controls.

The IDE model requires a Burgers vector or a slip plane in the matrix rather than in the precipitate. The investigation to HCP–BCC transformation will be described in two sections, i.e., BCC-to-HCP and HCP-to-BCC. The former will be addressed in detail, whereas the latter will be simply summarised with the results to avoid repeating in the context.

## 3. BCC-to-HCP Transformation

### 3.1. Lattice Correspondence and Initial OR

**Figure 2:**
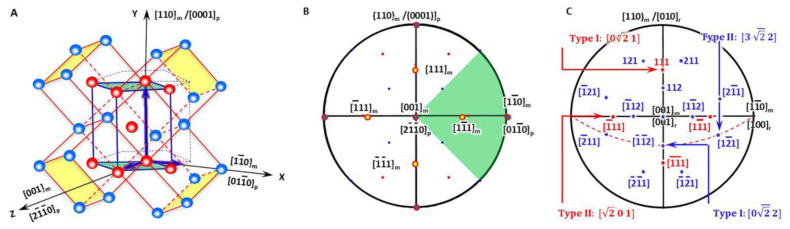
Lattice correspondence between HCP and BCC structures. (**A**) 3D atomic model. (**B**) Stereographic projection showing initial P-S OR describing lattice correspondence. (**C**) Burgers vector and shear plane variants in reference coordination system.

In a BCC-to-HCP phase transformation, it is accepted to choose the Pitsch–Schrader (P-S) lattice correspondence, which was reported in the precipitation of hexagonal *ε*-carbide from BCC ferrite by Pitsch and Schrader [18]. Accordingly, the diffusional phase transformation of BCC-to-HCP can be regarded as close-packed vector shearing at the close-packed plane. It is proposed as an initial OR where the close-packed plane of BCC is parallel to that of HCP and so is the close-packed direction. Figure 2A plots a three-dimensional crystal model highlighting how an HCP unit cell is constructed from four BCC unit cells. To make it clear, it is then re-plot as a two-dimensional stereographic projection circle in Figure 2B so that the orientation relationship between crystalline directions and planes of BCC and HCP crystals is straightforward. On the basis of Figure 2B, the variants of Burgers vectors that will be used for defining the nonrotated vector are shown in Figure 2C and will be discussed in Section 3.2.

As shown in Figure 2A, the generalised lattice corresponding between the BCC matrix (denoted as ‘_m_’) and HCP precipitate (denoted as ‘_p_’) described by P-S OR for the HCP–BCC system defines a reference coordination system O-XYZ and is expressed as

(9) $$\left\{\begin{array}{l} X:\;{\left[110\right]}_{m}//{\left[0001\right]}_{p}\\ Y:\;{\left[1\bar{1}0\right]}_{m}//{\left[01\bar{1}0\right]}_{p}\\ Z:\;{\left[001\right]}_{m}//{\left[2\bar{1}\bar{1}0\right]}_{p} \end{array}\right.$$

The reference coordinate system is referred to the BCC lattice coordination system. The Bain strain is determined by a directional ratio along each coordinate system axis between the matrix and product, where *γ_1_* = *a_b_/a_h_* and *γ_2_* = *c_h_/a_h_*, and the term ‘_b_’ (‘_h_’) denotes ‘BCC’ (‘HCP’); *a_b_*, *a_h_*, and *c_h_* separately stand for the lattice parameters of BCC and HCP. The two lattice parameter ratios (LPRs) are defined so that they are the same in both BCC-to-HCP and HCP-to-BCC transformations.

The initial lattice orientation relationship between the BCC matrix and HCP product is shown in the stereographic projection (Figure 2B), where four of the BCC Burgers vectors <111> are also highlighted with yellow colour solid circles.

As shown in Figure 2A, the Bain strain of a BCC-to-HCP transformation is

(10) $$B=\left(\begin{array}{ccc} \eta_{1} & 0 & 0\\ 0 & \eta_{2} & 0\\ 0 & 0 & \eta_{3} \end{array}\right)=\left(\begin{array}{ccc} \frac{c_{h}}{\sqrt{2}a_{b}} & 0 & 0\\ 0 & \frac{\sqrt{3}a_{h}}{\sqrt{2}a_{b}} & 0\\ 0 & 0 & \frac{a_{h}}{a_{b}} \end{array}\right)=\left(\begin{array}{ccc} \frac{\gamma_{2}}{\sqrt{2}\gamma_{1}} & 0 & 0\\ 0 & \sqrt{\frac{3}{2}}\frac{1}{\gamma_{1}} & 0\\ 0 & 0 & \frac{1}{\gamma_{1}} \end{array}\right)$$

Here, the parameters *η_1_*, *η_2_*, and *η_3_* are the three principle strains of the Bain strain, *γ*_1_ = *a_h_*/*a_b_*, and *γ*_2_ = *c_h_*/*a_b_*. The rotation matrix ***C***, which converts the BCC lattice to the reference system, is

(11) $$C=\left(\begin{array}{ccc} \frac{1}{\sqrt{2}} & -\frac{1}{\sqrt{2}} & 0\\ \frac{1}{\sqrt{2}} & \frac{1}{\sqrt{2}} & 0\\ 0 & 0 & 1 \end{array}\right)$$

### 3.2. Isothermal Precipitation

For an isothermal precipitation, the non-inclined vector in the direct space is the shortest Burgers vector in the matrix. The shortest Burgers vector in the BCC matrix is **b** = <111>_m_/2 and has four variants; see the four yellow solid circles in Figure 2B. Its form in the reference coordination system **b′** is obtained by

(12) $$b^{\prime }=Cb=\left(\begin{array}{ccc} \frac{1}{\sqrt{2}} & -\frac{1}{\sqrt{2}} & 0\\ \frac{1}{\sqrt{2}} & \frac{1}{\sqrt{2}} & 0\\ 0 & 0 & 1 \end{array}\right)\frac{1}{2}{\left[\begin{array}{c} 1\\ 1\\ 1 \end{array}\right]}_{m}=\frac{1}{2}{\left[\begin{array}{c} 0\\ \sqrt{2}\\ 1 \end{array}\right]}_{r}$$

The item ‘_r_’ is for the reference coordination system. All the four variants have a form $<0\frac{1}{\sqrt{2}}1]\;$in the reference coordinate system (the vector class <0vw] contains [0*vw*], $\left[0\bar{v}w\right]$, $\left[0v\bar{w}\right]$, $\left[0\bar{vw}\right]$, [*v*0*w*], $\left[\bar{v}0w\right]$, $\left[v0\bar{w}\right]$, and $\left[\bar{v}0\bar{w}\right]$; see Figure 2C). Hereafter, the following crystallographic calculation is carried out in the reference coordination system. Consequently, the non-inclined vector **P_1_** can be expressed as two types if the principal Bain strain along the *X*-axis is different from that along the *Y*-axis.

#### 3.2.1. Type I Burgers vector: ${\left[111\right]}_{m}=\frac{1}{2}{\left[0\sqrt{2}1\right]}_{r}$

When a Burgers vector in BCC matrix is chosen as [111], the non-rotated vector **P_1_** and the corresponding transformed vector **P_2_** has the following form:


(13) $$\left\{\begin{array}{c} P_{1}=\frac{b^{\prime }}{\left|b^{\prime }\right|}=\frac{1}{\sqrt{3}}\left[\begin{array}{c} 0\\ \sqrt{2}\\ 1 \end{array}\right]\\ P_{2}=\frac{BP_{1}}{|BP_{1}|}=\frac{\left(\begin{array}{ccc} \eta_{1} & 0 & 0\\ 0 & \eta_{2} & 0\\ 0 & 0 & \eta_{3} \end{array}\right)\frac{1}{\sqrt{3}}\left[\begin{array}{c} 0\\ \sqrt{2}\\ 1 \end{array}\right]}{\left|\left(\begin{array}{ccc} \eta_{1} & 0 & 0\\ 0 & \eta_{2} & 0\\ 0 & 0 & \eta_{3} \end{array}\right)\frac{1}{\sqrt{3}}\left[\begin{array}{c} 0\\ \sqrt{2}\\ 1 \end{array}\right]\right|}=\frac{1}{\sqrt{2\eta_{2}^{2}+\eta_{3}^{2}}}\left[\begin{array}{c} 0\\ \sqrt{2}\eta_{2}\\ \eta_{3} \end{array}\right]=\frac{1}{2}\left[\begin{array}{c} 0\\ \sqrt{3}\\ 1 \end{array}\right] \end{array}\right.$$

After Equation (1), a general formula for **Q_1_** has a form

(14) $$\left\{\begin{array}{l} Q_{1}=\left[\begin{array}{c} h\\ \bar{1}\\ \sqrt{2} \end{array}\right]\;\\ Q_{2}=B^{-1}Q_{1}=\left(\begin{array}{ccc} \frac{1}{\eta_{1}} & 0 & 0\\ 0 & \frac{1}{\eta_{2}} & 0\\ 0 & 0 & \frac{1}{\eta_{3}} \end{array}\right)\left[\begin{array}{c} h\\ \bar{1}\\ \sqrt{2} \end{array}\right]=\left[\begin{array}{c} \frac{h}{\eta_{1}}\\ -\frac{1}{\eta_{2}}\\ \frac{\sqrt{2}}{\eta_{3}} \end{array}\right] \end{array}\right.$$

Because invariant normal keeps its module unchanged (see Equation (2)), the to-be-determined variant ‘*h*’ is solved:

(15) $$h^{2}=\frac{3\eta_{2}^{2}\eta_{3}^{2}-2\eta_{2}^{2}-\eta_{3}^{2}}{1-\eta_{1}^{2}}\frac{\eta_{1}^{2}}{\eta_{2}^{2}\eta_{3}^{2}}=\frac{\gamma_{2}^{2}\left(9-8\gamma_{1}^{2}\right)}{3\left(2\gamma_{1}^{2}-\gamma_{2}^{2}\right)},\;\;\frac{\;\gamma_{2}-3/2}{\gamma_{1}-\sqrt{9/8}}>\sqrt{2}$$

**Figure 3:**
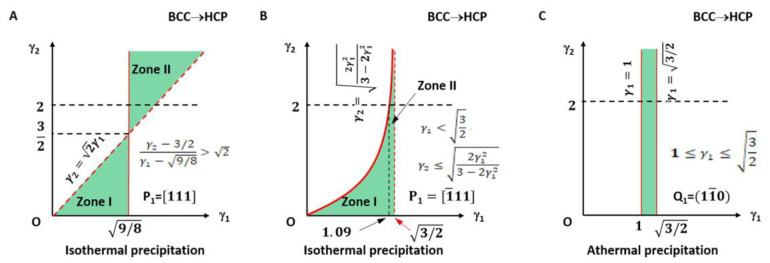
Lattice parameter applicable range (green colour) for BCC-to-HCP transformation of isothermal (**A**,**B**) and athermal (**C**) precipitation. (**A**) Triangular zones I and II for isothermal type I. (**B**) Right side of quadratic curve for isothermal type II. Dashed line indicates forbidden line. (**C**) Rectangle zone of athermal type.

The applicable LPR for type I is plotted in Figure 3A and highlighted with light green colour. It falls in the small fractions of the LPR in zone I and zone II.

#### 3.2.2. Type II Burgers vector: $\frac{1}{2}\left[\sqrt{2}01\right]$

For the case in which the LPR does not meet the condition in Equation (15), an alternative **P**_1_ should be chosen.


(16) $$\left\{\begin{array}{c} P_{1}=\frac{b^{\prime }}{\left|b^{\prime }\right|}=\frac{1}{\sqrt{3}}\left[\begin{array}{c} \sqrt{2}\\ 0\\ 1 \end{array}\right]\\ P_{2}=\frac{BP_{1}}{\left|BP_{1}\right|}=\frac{\left(\begin{array}{ccc} \eta_{1} & 0 & 0\\ 0 & \eta_{2} & 0\\ 0 & 0 & \eta_{3} \end{array}\right)\frac{1}{\sqrt{3}}\left[\begin{array}{c} \sqrt{2}\\ 0\\ 1 \end{array}\right]}{\left|\left(\begin{array}{ccc} \eta_{1} & 0 & 0\\ 0 & \eta_{2} & 0\\ 0 & 0 & \eta_{3} \end{array}\right)\frac{1}{\sqrt{3}}\left[\begin{array}{c} \sqrt{2}\\ 0\\ 1 \end{array}\right]\right|}=\frac{1}{\sqrt{2\eta_{1}^{2}+\eta_{3}^{2}}}\left[\begin{array}{c} \sqrt{2}\eta_{1}\\ 0\\ \eta_{3} \end{array}\right]=\frac{1}{\sqrt{\gamma_{2}^{2}+1}}\left[\begin{array}{c} \gamma_{2}\\ 0\\ 1 \end{array}\right] \end{array}\right.$$

The invariant normal **Q_1_** is obtained in the same way.

(17) $$\left\{\begin{array}{l} Q_{1}=\left[\begin{array}{c} \bar{1}\\ k\\ \sqrt{2} \end{array}\right]\;\\ Q_{2}=B^{-1}Q_{1}=\left(\begin{array}{ccc} \frac{1}{\eta_{1}} & 0 & 0\\ 0 & \frac{1}{\eta_{2}} & 0\\ 0 & 0 & \frac{1}{\eta_{3}} \end{array}\right)\left[\begin{array}{c} \bar{1}\\ k\\ \sqrt{2} \end{array}\right]=\left[\begin{array}{c} -\frac{1}{\eta_{1}}\\ \frac{k}{\eta_{2}}\\ \frac{\sqrt{2}}{\eta_{3}} \end{array}\right] \end{array}\right.$$

Moreover, the variant ‘*k*’ follows

(18) $$k^{2}=\frac{3\eta_{1}^{2}\eta_{3}^{2}-2\eta_{1}^{2}-\eta_{3}^{2}}{1-\eta_{2}^{2}}\frac{\eta_{2}^{2}}{\eta_{1}^{2}\eta_{3}^{2}}=\frac{3}{\gamma_{2}^{2}}\frac{2\gamma_{1}^{2}-\gamma_{2}^{2}\left(3-2\gamma_{1}^{2}\right)}{3-2\gamma_{1}^{2}},\;\gamma_{1}<\sqrt{\frac{3}{2}}\;\&\;\gamma_{2}\leq \sqrt{\frac{2\gamma_{1}^{2}}{3-2\gamma_{1}^{2}}}\;Or\;\gamma_{1}>\sqrt{\frac{3}{2}}\;\&\;\gamma_{2}\geq \sqrt{\frac{2\gamma_{1}^{2}}{3-2\gamma_{1}^{2}}}$$

The lattice parameter applicable range of the above two types of a non-inclined vector is shown in Figure 3B.

It can be seen in Figure 3A that the parameters *γ_1_* and *γ_2_* for type I are not arbitrarily applicable. They fall into the green colour triangular zones only. Moreover, those for type II are valid only on the right side of the quadratic curve. The dashed line indicates the forbidden line.

### 3.3. Athermal Precipitation

A shear is required for athermal precipitation, though it is not necessary to produce an invariant plane normal in the shear direction. The only condition is the orientation of shear plane **Q_1_**. This condition is enough to find a one-step rotation solution to generate the invariant line strain because a non-inclined plane must contain an invariant line **P_1_**.

According to the lattice correspondence of BCC-to-HCP transformation, the shear happening in the BCC matrix possesses (110) <1–11> shear deformation mechanism. The shear plane keeps non-inclined while there exists an invariant line in the shear plane. However, the shear direction may alter and diverse in various directions to accommodate deformation because there is no macroscopy deformation. Consequently, the controlling items are the shear plane normal **Q_1_** and the invariant line in the shear plane **P_1_**.

In the reference coordination system, the shear plane **s′** has two forms, i.e., **s_1_′** and **s_2_′**, which are obtained by a rotation operation for each of the type **s** = {110}(**s_1_ =** $\left(1\bar{1}0\right)$, **s_2_ =** $\left(0\bar{1}1\right)$):

Type I shear plane: $\left(1\bar{1}0\right)$

(19) $${s_{1}}^{\prime }=s_{1}C^{-1}=\left(1\bar{1}0\right)\left(\begin{array}{ccc} \frac{1}{\sqrt{2}} & \frac{1}{\sqrt{2}} & 0\\ -\frac{1}{\sqrt{2}} & \frac{1}{\sqrt{2}} & 0\\ 0 & 0 & 1 \end{array}\right)=\left[\begin{array}{c} \sqrt{2}\\ 0\\ 0 \end{array}\right]$$

Type II shear plane:$\;\left(0\bar{1}1\right)$

(20) $${s_{2}}^{\prime }=s_{2}C^{-1}=\left(0\bar{1}1\right)\left(\begin{array}{ccc} \frac{1}{\sqrt{2}} & \frac{1}{\sqrt{2}} & 0\\ -\frac{1}{\sqrt{2}} & \frac{1}{\sqrt{2}} & 0\\ 0 & 0 & 1 \end{array}\right)=\frac{1}{\sqrt{2}}\left[\begin{array}{c} \bar{1}\\ 1\\ \sqrt{2} \end{array}\right]$$

However, only type I can be used because the second type$\;\left(0\bar{1}1\right)$ is not parallel to (0001).

(21) $$\left\{\begin{array}{l} Q_{1}=\frac{s_{1}\prime }{\left|s_{1}\prime \right|}=\left[\begin{array}{c} 1\\ 0\\ 0 \end{array}\right]\;\\ Q_{2}=\frac{B^{-1}P_{1}}{\left|B^{-1}P_{1}\right|}=\left[\begin{array}{c} 1\\ 0\\ 0 \end{array}\right] \end{array}\right.$$

There is an invariant line **Q_1_** in the shear plane **s′**. It has a form $\left[0\bar{w}v\right]$.

(22) $$\left\{\begin{array}{l} P_{1}=\left[uvw\right]\\ P_{2}=BP_{1}=\left(\begin{array}{ccc} \eta_{1} & 0 & 0\\ 0 & \eta_{2} & 0\\ 0 & 0 & \eta_{3} \end{array}\right)\left[\begin{array}{c} u\\ v\\ w \end{array}\right] \end{array}\right.$$

under the constraint

(23) $$\left\{\begin{array}{l} P_{1}\cdot s_{1}{}^{\prime }=0\\ \left|P_{2}\right|=\left|P_{1}\right| \end{array}\right.$$

It is immediately used to obtain the invariant line **P_1_** from the above equation:

(24) $$\left\{\begin{array}{l} P_{1}=\left[0\;\sqrt{2\gamma_{1}^{2}-2}\;\sqrt{3-2\gamma_{1}^{2}}\right]\\ P_{2}=\left[0\;\frac{\sqrt{3\left(2\gamma_{1}^{2}-2\right)}}{\sqrt{2}\gamma_{1}}\;\frac{\sqrt{3-2\gamma_{1}^{2}}}{\gamma_{1}}\right] \end{array},\;1\leq \gamma_{1}\leq \sqrt{\frac{3}{2}}\right.$$

The obtained parameters are induced to Equation (3) so that a rotation operation is resolved. The LPR range is shown in Figure 3C. The rotation axis is parallel to **Q_1_** as **Q_1_** is parallel to **Q_2_**. The rotation angle is defined by the included angle between **P_1_** and **P_2_**:

(25) $$cos\theta =\frac{\left(\sqrt{3}-\sqrt{2}\right)\left(\sqrt{2}\gamma_{1}^{2}+\sqrt{3}\right)}{\gamma_{1}}$$

It is not strange that this result is not altered by *γ*_2_ and so only the limit of *γ*_1_ is practical.

## 4. HCP-to-BCC Transformation

### 4.1. Lattice Correspondence and Initial OR

**Figure 4:**
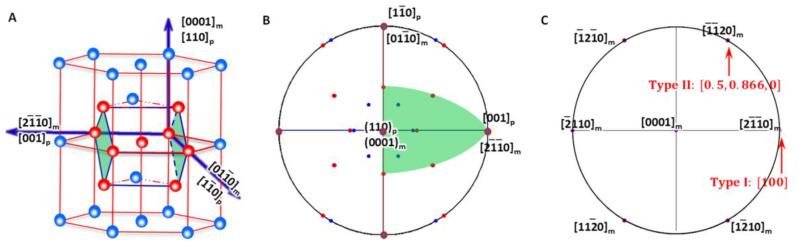
Lattice correspondence between HCP and BCC structures. (**A**) 3D atomic model showing how to obtain a BCC lattice from two HCP unit cells. (**B**) Stereographic projection showing the initial P-S OR corresponding to lattice correspondence. (**C**) Burgers vector and shear plane variants in reference coordination system.

As shown in Figure 4A, the generalised lattice corresponding to HCP-to-BCC transformation described by P-S OR (Figure 4B) defines a reference coordination system O-XYZ and is expressed as

(26) $$\left\{\begin{array}{l} X:\;{\left[2\bar{1}\bar{1}0\right]}_{h}//{\left[001\right]}_{b}\\ Y:\;{\left[01\bar{1}0\right]}_{h}//{\left[1\bar{1}0\right]}_{b}\\ Z:\;{\left[0001\right]}_{h}//{\left[110\right]}_{b} \end{array}\right.$$

Compared with that for BCC-to-HCP transformation, the lattice correspondence is identical. However, by using the HCP lattice as the reference coordination system, the Burgers vectors for the matrix are now one of the three basal vectors, i.e., $\left[2\bar{1}\bar{1}0\right]$,$\;\left[\bar{1}2\bar{1}0\right]$, and $\left[\bar{1}\bar{1}20\right]$. The Bain strain is

(27) $$B=\left(\begin{array}{ccc} \eta_{1} & 0 & 0\\ 0 & \eta_{2} & 0\\ 0 & 0 & \eta_{3} \end{array}\right)=\left(\begin{array}{ccc} \frac{a_{b}}{a_{h}} & 0 & 0\\ 0 & \frac{\sqrt{2}a_{b}}{\sqrt{3}a_{h}} & 0\\ 0 & 0 & \frac{\sqrt{2}a_{b}}{c_{h}} \end{array}\right)=\left(\begin{array}{ccc} \gamma_{1} & 0 & 0\\ 0 & \sqrt{\frac{2}{3}}\gamma_{1} & 0\\ 0 & 0 & \frac{\sqrt{2}\gamma_{1}}{\gamma_{2}} \end{array}\right)$$

where *γ_1_* = *a_b_/a_h_* and *γ_2_* = *c_h_/a_h_*. This Bain strain is slightly different in the order of the three principal strains as that given in Equation (10).

The rotation matrix ***C***, which converts the HCP lattice into the reference orthogonal coordination system, is

(28) $$C=\left(\begin{array}{ccc} 1 & 0 & 0\\ 0 & 1 & 0\\ 0 & 0 & 1 \end{array}\right)$$

This is an identity matrix, and, consequently, no conversion needs to be applied after the crystallographic calculation of OR, IL, and HP.

### 4.2. Isothermal Precipitation

The non-inclined vector in the direct space is the shortest Burgers vector in the basal plane of the HCP matrix. The shortest Burgers vector in the HCP matrix is $V_{h}=\left[2\bar{11}0\right]$ and has six variants. Its form in the reference coordination system is obtained by

(29) $$P_{1}=CV_{b}=\left(\begin{array}{ccc} 1 & 0 & 0\\ 0 & 1 & 0\\ 0 & 0 & 1 \end{array}\right){\left[\begin{array}{c} 1\\ 0\\ 0 \end{array}\right]}_{h}={\left[\begin{array}{c} 1\\ 0\\ 0 \end{array}\right]}_{r}$$

In the three-index orthogonal reference coordinate system, the variants of the HCP Burgers vector $\left[2\bar{11}0\right]$ have two forms, $\left[\pm 100\right]$, $\left[-\frac{1}{2},\pm \frac{\sqrt{3}}{2},0\right]$ and $\left[\frac{1}{2},\pm \frac{\sqrt{3}}{2},0\right]$ (see Figure 4C). Hereafter, the following crystallographic calculation is carried out in this coordinate system. Consequently, the non-inclined vector **P_1_** can be expressed as two types if the principal Bain strain along the *X*-axis is different from that along the *Y*-axis.

#### 4.2.1. Type I: $\left[2\bar{11}0\right]//\left[100\right]$

When a Burgers vector in HCP matrix is chosen as $\left[2\bar{11}0\right]$, the non-rotated vector **P_1_** and the corresponding transformed vector **P_2_** has the following form:

(30) $$\left\{\begin{array}{l} P_{1}=\left[\begin{array}{c} 1\\ 0\\ 0 \end{array}\right]\;\\ P_{2}=\frac{BP_{1}}{\left|BP_{1}\right|}=\frac{\left(\begin{array}{ccc} \eta_{1} & 0 & 0\\ 0 & \eta_{2} & 0\\ 0 & 0 & \eta_{3} \end{array}\right)\left[\begin{array}{c} 1\\ 0\\ 0 \end{array}\right]}{\left|\left(\begin{array}{ccc} \eta_{1} & 0 & 0\\ 0 & \eta_{2} & 0\\ 0 & 0 & \eta_{3} \end{array}\right)\left[\begin{array}{c} 1\\ 0\\ 0 \end{array}\right]\right|}=\left[\begin{array}{c} 1\\ 0\\ 0 \end{array}\right] \end{array}\right.$$

After Equation (1), a general formula for **Q_1_** has a form

(31) $$\left\{\begin{array}{l} Q_{1}=\left[\begin{array}{c} 0\\ k\\ l \end{array}\right],k^{2}+l^{2}=1\;\\ Q_{2}=B^{-1}Q_{1}=\left(\begin{array}{ccc} \frac{1}{\eta_{1}} & 0 & 0\\ 0 & \frac{1}{\eta_{2}} & 0\\ 0 & 0 & \frac{1}{\eta_{3}} \end{array}\right)\left[\begin{array}{c} 0\\ k\\ l \end{array}\right]=\left[\begin{array}{c} 0\\ \frac{k}{\eta_{2}}\\ \frac{l}{\eta_{3}} \end{array}\right] \end{array}\right.$$

Because invariant normal keeps it module-invariant (see Equation (2)), the to-be-determined variant ‘*k*’ and ‘*l*’ is solved:

(32) $$k^{2}=\frac{\eta_{2}^{2}\left(\eta_{3}^{2}-1\right)}{\eta_{3}^{2}-\eta_{2}^{2}}=\frac{\gamma_{2}^{2}-2\gamma_{1}^{2}}{\gamma_{2}^{2}-3},\;\;l^{2}=\frac{\eta_{3}^{2}\left(1-\eta_{2}^{2}\right)}{\eta_{3}^{2}-\eta_{2}^{2}}=\frac{2\gamma_{1}^{2}-3}{\gamma_{2}^{2}-3},\;\frac{\;\gamma_{2}-\sqrt{3}}{\left(\gamma_{1}-\sqrt{3/2}\right)}>\sqrt{2}$$

**Figure 5:**
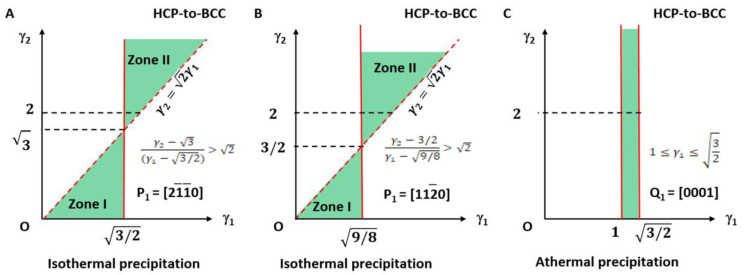
Lattice parameter applicable range (green colour) for HCP-to-BCC transformation of isothermal (**A**,**B**) and athermal (**C**) precipitation. (**A**) Triangular zones I and II for isothermal type I. (**B**) Triangular zones I and II for isothermal type II. Dashed line indicates forbidden line. (**C**) Rectangle zone of athermal type.

Obviously, this is degraded to the 2D case of an invariant line strain model. The applicable LPR for type I can be summarised as the equation given in Equation (32) and is plotted in Figure 5A. It falls in the small fractions of the LPR in zone I and zone II and highlighted with light green colour.

Since **P_2_** is parallel to **P_1_**, the rotation axis will be parallel to any of **P_2_** or **P_1_,** and the rotation angle *θ* is the included angle between **Q_1_** and **Q_2_**. This gives the solution of the rotation operation straightforward:

(33) $$u=\left[100\right],\;cos\theta =\frac{1+\eta_{2}\eta_{3}}{\eta_{2}+\eta_{3}}$$

#### 4.2.2. Type II: $\left[11\bar{2}0\right]//\left[\frac{1}{2},\frac{\sqrt{3}}{2},0\right]$

For the case in which the LPR does not meet the condition in Equation (15), an alternative **P**_1_ should be chosen.


(34) $$\left\{\begin{array}{c} P_{1}=\left[\begin{array}{c} \frac{1}{2}\\ \frac{\sqrt{3}}{2}\\ 0 \end{array}\right]\\ P_{2}=\frac{BP_{1}}{\left|BP_{1}\right|}=\frac{\left(\begin{array}{ccc} \eta_{1} & 0 & 0\\ 0 & \eta_{2} & 0\\ 0 & 0 & \eta_{3} \end{array}\right)\left[\begin{array}{c} \frac{1}{2}\\ \frac{\sqrt{3}}{2}\\ 0 \end{array}\right]}{\left|\left(\begin{array}{ccc} \eta_{1} & 0 & 0\\ 0 & \eta_{2} & 0\\ 0 & 0 & \eta_{3} \end{array}\right)\left[\begin{array}{c} \frac{1}{2}\\ \frac{\sqrt{3}}{2}\\ 0 \end{array}\right]\right|}=\frac{2}{\sqrt{\eta_{1}^{2}+3\eta_{2}^{2}}}\left[\begin{array}{c} \frac{\eta_{1}}{2}\\ \frac{\sqrt{3}\eta_{2}}{2}\\ 0 \end{array}\right]=\frac{1}{\sqrt{3}}\left[\begin{array}{c} 1\\ \sqrt{2}\\ 0 \end{array}\right] \end{array}\right.$$

Then, the invariant normal **Q_1_** can be solved.

(35) $$\left\{\begin{array}{l} Q_{1}=\left[\begin{array}{c} \sqrt{3}\\ \bar{1}\\ l \end{array}\right]\;\\ Q_{2}=B^{-1}Q_{1}=\left(\begin{array}{ccc} \frac{1}{\eta_{1}} & 0 & 0\\ 0 & \frac{1}{\eta_{2}} & 0\\ 0 & 0 & \frac{1}{\eta_{3}} \end{array}\right)\left[\begin{array}{c} \sqrt{3}\\ \bar{1}\\ l \end{array}\right]=\left[\begin{array}{c} \frac{\sqrt{3}}{\eta_{1}}\\ \frac{-1}{\eta_{2}}\\ \frac{l}{\eta_{3}} \end{array}\right] \end{array}\right.$$

In addition, the solved variant ‘*l*’ follows:

(36) $$l^{2}=\frac{4\eta_{1}^{2}\eta_{2}^{2}-3\eta_{2}^{2}-\eta_{1}^{2}}{1-\eta_{3}^{2}}\frac{\eta_{3}^{2}}{\eta_{1}^{2}\eta_{2}^{2}}=\frac{9-8\gamma_{1}^{2}}{2\gamma_{1}^{2}-\gamma_{2}^{2}},\;\;\frac{\;\gamma_{2}-3/2}{\gamma_{1}-\sqrt{9/8}}>\sqrt{2}\;$$

The lattice parameter applicable range of the above two types of a non-inclined vector is shown in Figure 5B.

### 4.3. Athermal Precipitation

According to the lattice correspondence of the HCP-to-BCC transformation, the shear happening in the HCP matrix possesses the deformation system (0001)$<\bar{1}\bar{1}20>$. The controlling items are the shear plane normal **Q_1_** and the invariant line in the shear plane **P_1_**.

In the reference coordination system, the shear plane **s′** has only one form, which is the same as that in the HCP lattice system, which is used as the reference coordination system. **s** = (001):

(37) $$s=\left[\begin{array}{c} 0\\ 0\\ 1 \end{array}\right]$$

Similar with isothermal precipitation, the non-inclined vector **Q_1_** can be expressed as

(38) $$\left\{\begin{array}{l} Q_{1}=\frac{s_{1}\prime }{\left|s_{1}\prime \right|}=\left[\begin{array}{c} 0\\ 0\\ 1 \end{array}\right]\;\\ Q_{2}=\frac{B^{-1}P_{1}}{\left|B^{-1}P_{1}\right|}=\left[\begin{array}{c} 0\\ 0\\ 1 \end{array}\right] \end{array}\right.$$

There is an invariant line **P_1_** in the shear plane **s′**. It has a form [*uv*0].

(39) $$\left\{\begin{array}{l} P_{1}=\left[uvw\right]\\ P_{2}=BP_{1}=\left(\begin{array}{ccc} \eta_{1} & 0 & 0\\ 0 & \eta_{2} & 0\\ 0 & 0 & \eta_{3} \end{array}\right)\left[\begin{array}{c} u\\ v\\ w \end{array}\right] \end{array}\right.$$

under the constraint

(40) $$\left\{\begin{array}{l} P_{1}\cdot s_{1}{}^{\prime }=0\\ \left|P_{2}\right|=\left|P_{1}\right| \end{array}\right.$$

It is immediately used to obtain the invariant line **P_1_** from the above equation:

(41) $$\left\{\begin{array}{l} P_{1}=\left[\frac{\sqrt{3-2\gamma_{1}^{2}}}{\gamma_{1}}\;\frac{\sqrt{3\gamma_{1}^{2}-3}}{\gamma_{1}}\;0\right]\\ P_{2}=\left[\;\sqrt{3-2\gamma_{1}^{2}}\;\sqrt{2\gamma_{1}^{2}-2}\;0\right] \end{array},\;1\leq \gamma_{1}\leq \sqrt{\frac{3}{2}}\right.$$

The rotation axis is parallel to **Q_1_** as **Q_1_** is parallel to **Q_2_**. The rotation angle is defined by the included angle between **P_1_** and **P_2_**:

(42) $$cos\theta =\frac{\left(3-\sqrt{6}\right)+\gamma_{1}^{2}\left(\sqrt{6}-2\right)}{\gamma_{1}}$$

The LPR range is shown in Figure 5C. It is found again that this result is not altered by *γ*_2_ and so only the limit of *γ*_1_ is necessary.

## 5. Evaluation of the Predicted Results for HCP–BCC Transformation Systems

Five examples of diffusional phase transformations of the HCP–BCC system are investigated using the IDE model to evaluate its applicability.

Three isothermal precipitations are *α*-Mg⟶*γ*-Mg_17_Al_12_, *α*-Zr⟶*β*-Nb, and *α*-Mo⟶Mo_2_C [19]. Two athermal precipitations are *β*-Ti⟶*α*-Ti, and *β*-Li⟶*α*-Mg [20]. The lattice parameters of the matrix and precipitate for each of the system are shown in Table 1. For the convenience of comparison, the three principal Bain strains of each precipitation system are also presented in the last second column of Table 2. For isothermal precipitations, the non-inclined direction is either [0001] for HCP or [110] for BCC. For athermal reactions, the invariant normal corresponds to the shear plane. The three principle strains for each of the transformation are all close to a unitary value. It can be also seen that these values are actually close to each other, which means the Bain strain corresponding to the HCP–BCC lattice reconstruction is practically very small. Accordingly, the calculated rotation angle of the zero-strain rotation is far less than 10°, which benefits in minimising the overall transformation energy.

The intermediate parameters for finding the one-step rotation axis, **P_1_, P_2_**, **Q_1_,** and **Q_2_,** are shown in Table 2. The isothermal precipitation system adopts the non-inclined vector **P_1_** parallel to the matrix’s Burgers vector, whereas the athermal one takes the non-inclined plane **Q_1_**. Since the calculation is carried out in the reference system, the items are also listed with their forms in the matrix system, which can be achieved by applying lattice correspondence transformation.

The calculation results of all the five investigated systems indicate a very small one-step rotation angle along a principal axis to accommodate the strain generated by lattice reconstruction. This is in coincidence with the fact that all the three principal Bain strains are very small. Consequently, the final OR between the two phases is very close to the initial OR and defined by the Burgers OR.

It is then convenient to derive eigenvalues, eigenvectors, and eigenplanes from the total strain matrix ***A*** = ***RB***. Table 3 presents the calculated crystallographic features of the five precipitation systems including OR, HP, and IL. To evaluate the calculated results, experimental data are shown in the adjacent column accompanied with the discrepancy angle between the calculated ones and experimental results.

The angle deviation in this table is less than 5°. Given that the measurement accuracy of the crystallographic vector and plane normal by conventional electron diffraction under TEM has a tolerance of about ±5°, it can be claimed with confidence that the calculated accuracy falls into this tolerance and is convincing. The IDE model for HCP–BCC precipitation systems has now shown its effectiveness after the investigation of the above five systems.

It is also found that the calculated crystallographic features in the current work are very close to those in other reported work as shown in the extreme right column of Table 3. Of course, multiple rotation operations were applied in their models, whereas the current work applied one-step rotation only.

The comparison between the calculated values and experimental data and reported work showed that the IDE model is capable of deriving most of the precipitate’s OR, HP, and IL of HCP–BCC isothermal and athermal precipitation systems. On the basis of the above success, the next section will be extended to a systematic investigation of the complicated HCP–BCC precipitation system containing two LPR variants.

## 6. Systematic Demonstration of Predicted Results for HCP–BCC Systems in Full LPR Range

The application of the IDE model to the HCP–BCC transformation system has been verified in the above-mentioned five systems including the *α*-Mg⟶*γ*-Mg_17_Al_12_ precipitation system. This work will further explore a global pattern of crystallographic features in a full LPR range, which have not been seen in the literature yet.

To make the calculation of the predicted crystallographic features in a satisfied speed with acceptable precision, the current author developed a desktop Window program and then completed all the calculations and plotting by using the algorithm shown in this work. The program named “SP3 for CPT” is composed by using Visual Basic 6.0 and capable of running on a Windows operating system. The basic function is to make full use of composited stereographic projection to visualise crystallographic features of solid-state phase transformations covering diffusional and martensitic transformation. The crystallographic features are calculated by using the invariant deformation element (IDE) model, which was also developed by the current author [14]. It will be submitted to some journal as an independent technical paper to let the program be publically accessible. For the convenience of comparison of the calculated crystallographic features, the results are shown for both HCP-to-BCC and BCC-to-HCP transformations and plotted in stereographic projections sharing the same pole centre [001].

**Figure 6:**
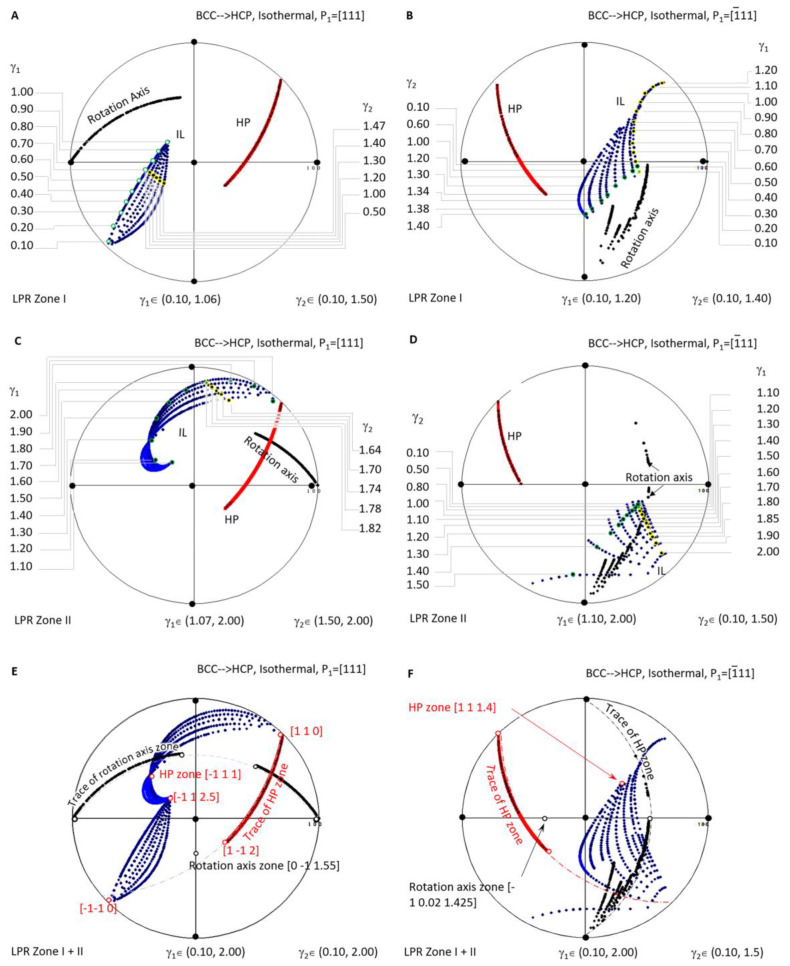
Stereographic project showing calculated crystallographic features (HP and IL) of BCC-to-HCP precipitation under the condition of non-inclined vector parallel to BCC Burgers vector. (**A**) Isothermal LPR Zone I for [111]. (**B**) Isothermal LPR Zone II for [111]. (**C**) Isothermal LPR Zone I for $\left[\bar{1}11\right]$. (**D**) Isothermal LPR Zone II for $\left[\bar{1}11\right]$. (**E**) Overlapping of two LPR zones for [111]. (**F**) Overlapping of two LPR zones for $\left[\bar{1}11\right]$.

Figure 6 shows a systematic investigation of the crystallographic features (IL and HP) of BCC-to-HCP diffusional phase transformations for the case of isothermal precipitation controlled by the non-inclined vector **P_1_**//[111]. Figure 6A,C are the stereographic projections of the predicted HP and IL of LPR zone I where *γ*_2_ belongs to (0.10, 1.06) and *γ*_1_ to (0.10, 1.50), and LPR zone II where *γ*_2_ belongs to (1.07, 2.00) and *γ*_1_ to (1.50, 2.00). Figure 6B,D correspond to the case of **P_1_**//$\left[\bar{1}11\right]$. In detail, Figure 6B,D are the stereographic projections of the predicted HP and IL of LPR zone I where *γ*_2_ belongs to (0.1, 1.40) and *γ*_1_ to (0.1, 1.2), and LPR zone II where *γ*_2_ belongs to (0.1, 1.5) and *γ*_1_ to (1.10, 2.00). A combination stereographic projection showing the full lattice parameter ratio range is presented in Figure 6E for the case of Burgers vector P_1_//[111] and Figure 6F for **P_1_**//$\left[\bar{1}11\right]$, where *γ*_2_ belongs to (0.10, 2.00) and *γ*_1_ to (0.10, 2.00). In each of the six stereographic projections, a serial of dots for the invariant line (blue), habit plane (red), and rotation axis (black) are all plotted with colour visible in the online version.

When overlapping the two zones of the case **P_1_**//[111] in Figure 6E, it is found that all the rotation axes lie on the trace of $\left[0\bar{1}1.55\right]$, and all the HP poles belong to the same zone axis $\left[\bar{1}11\right]$. At the same time, IL points diverge into a narrow band and could not be assigned to the trace of a single plane. The case **P_1_**//[111], as shown in Figure 6F, exhibits a similar distribution of the rotation axis (some in the plane $\left(\bar{1}\;0.02\;1.425\right)$), HP (on the zone axis [1 1 1.4]), and IL (scattering a larger angle range).

A few interesting findings are highlighted in the above systematic stereographic projection investigation of the crystallographic features distribution of isothermal precipitation in BCC-to-HCP diffusional transformation. First of all, no matter the kind of the lattice parameter ratio of *γ*_1_ or *γ*_2_, the habit plane always belongs to a single zone axis, i.e., $\left[\bar{1}11\right]$ for **P_1_**//[111] and [1 1 1.4] for **P_1_**//$\left[\bar{1}11\right]$. Second, the growth direction or invariant line of the isothermal precipitate varies in a wide range of orientation, which is helpful for tuning the growth direction within the limitation of the habit plane. Finally, the zero-strain rotation axis is limited into a single plane $\left(01\bar{1.55}\right)$ for **P_1_**//[111] but diverges in a narrow band of planes for **P_1_**//$\left[\bar{1}11\right]$ (Figure 6).

**Figure 7:**
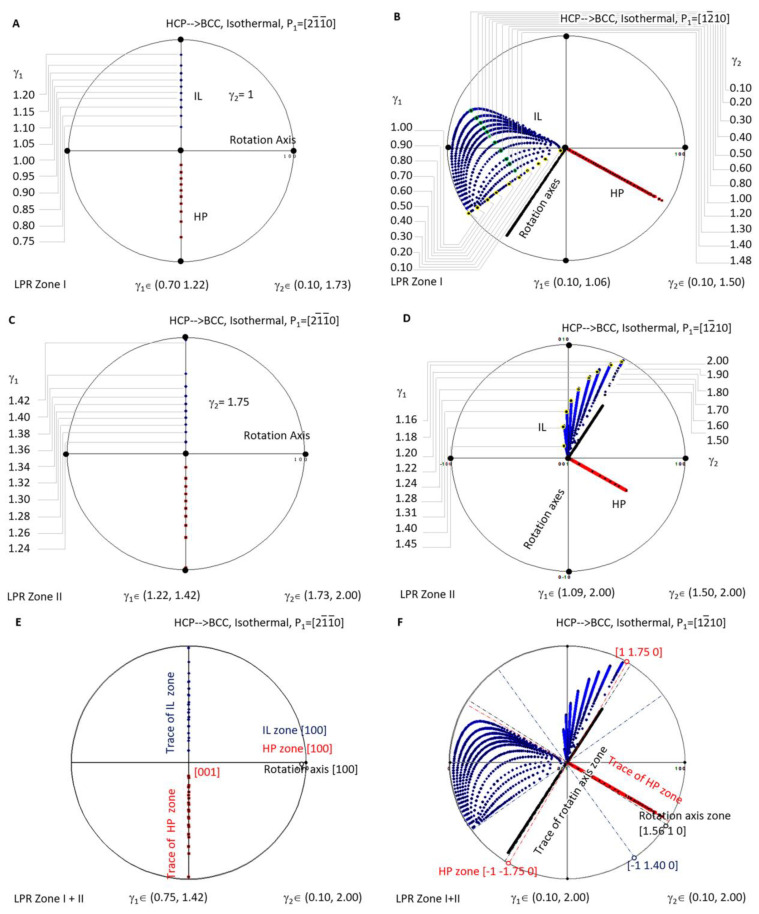
Stereographic project showing calculated crystallographic features (HP and IL) of HCP-to-BCC precipitation under the condition of non-inclined vector **P_1_** parallel to HCP Burgers vector. (**A**) Isothermal LPR Zone I for $\left[2\bar{11}0\right]$. (**B**) Isothermal LPR Zone II for $\left[2\bar{11}0\right]$. (**C**) Isothermal LPR Zone I for $\left[1\bar{2}10\right]$. (**D**) Isothermal LPR Zone II for $\left[1\bar{2}10\right]$. (**E**) Overlapping of two LPR zones for $\left[2\bar{11}0\right]$. (**F**) Overlapping of two LPR zones for $\left[1\bar{2}10\right]$.

A similar investigation was carried out for isothermal precipitation in HCP-to-BCC transformation. Figure 7A is a stereographic projection showing calculated HP and IL for isothermal precipitation controlled by a non-inclined vector parallel to $\left[2\bar{11}0\right]$ in LPR Zone I where *γ*_2_ belongs to (0.10, 1.73) and *γ*_1_ to (0.70, 1.22), and in Zone II where *γ*_2_ belongs to (1.73, 2.00) and *γ*_1_ to (1.22, 1.42). Figure 7C is a stereographic projection showing calculated HP and IL for isothermal precipitation controlled by a non-inclined vector parallel to $\left[\bar{1}2\bar{1}0\right]$ in LPR Zone I where *γ*_2_ belongs to (0.10, 1.50) and *γ*_1_ to (0.10, 1.06), and in Zone II where *γ*_2_ belongs to (1.50, 2.00) and *γ*_1_ to (1.09, 2.00). The Burgers vector and slip plane normal of the HCP structure are parallel to one of the three basal axes of the reference coordination system, so it is a one-step rotation axis. Consequently, the generated HP normal and IL locate at the same plane when the lattice parameter ratio *γ*_2_ varies. If HP and IL points are all plotted for each of *γ*_2_, they will occupy the whole trace of the plane $\left(2\bar{1}\bar{1}0\right)$ and become not distinguishable. For the sake of easy reading, the dataset is plotted for only one value of *γ*_2_, i.e., *γ*_2_ = 1 in Figure 7A and *γ*_2_ = 1.75 in Figure 7C. Figure 7B,D correspond to the case of **P_1_**//$\left[1\bar{2}10\right]$.

When overlapping the two LPR zones of the case **P_1_**//$\left[2\bar{11}0\right]$ as shown in Figure 7E, it is found that all the rotation axes are parallel to [100], and all the HP poles belong to the same zone [100]. At the same time, IL points lie on the trace of [100]. The case **P_1_**//$\left[1\bar{2}10\right]$, as shown in Figure 7F, shows a complicated distribution pattern of IL. However, that of the rotation axis (in the plane (1.56 1 0)) and that of HP (on the zone axis $\left[1\bar{1.75}\;0\right]$) still fall into a certain plane.

This indicates that, when the matrix of the isothermal precipitation in the HCP–BCC system is changed from BCC to HCP, the distribution of the crystallographic features becomes simplified. For instance, as mentioned in the above context, as long as the non-inclined vector **P_1_** is parallel to the principle axis of the reference coordination system, i.e., **P_1_**//[100], the rotation axis can only be this axis, and all the HP and IL fall into the trace of this axis. When the non-inclined vector **P_1_** is chosen not parallel to any principle axis, the rotation axis scatters in the plane (1.56 1 0) a few degrees away from $\left(1\bar{1}\;00\right)$, and the HP scatters in a zone axis $\left[\bar{1}\bar{1.75}\;0\right]$ equivalent to $\left[\bar{1}\bar{1}2\;0\right]$. At the same time, the invariant line diverges in a much larger orientation space that cannot be enclosed by limited planes. The second case offers more free space to accommodate transformation strain and is expected to be easily observed in many isothermal HCP-to-BCC diffusional transformations when crystal structures are far more complicated than a simple unit cell.

**Figure 8:**
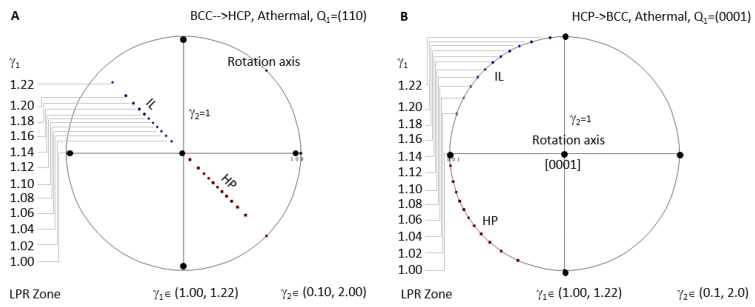
Stereographic project showing calculated crystallographic features (HP and IL) of athermal precipitation in HCP–BCC system. (**A**) BCC-to-HCP LPR zone under the condition of non-inclined vector **Q_1_** parallel to the close-packed plane (110). (**B**) HCP-to-BCC athermal LPR zone under the condition of non-inclined vector **Q_1_** parallel to basal plane (0001).

Figure 8A shows the case for BCC-to-HCP athermal precipitation where LPR *γ*_2_ has no limitation. Since all the IL spots fall in the same location when LPR *γ*_2_ changes, an arbitrary value between 0.1 and 2 is selected to show the distribution of IL and HP for different LPR *γ*_1_. HCP-to-BCC athermal precipitation is plotted in Figure 8B.

A few interesting outcomes can be obtained from these figures:

(1) No matter which LPR or which transformation direction, the one-step rotation axis always belongs to the same plane, whereas HP always shares the same zone axis, BCC [11*w*] (‘*w*’ maybe zero) or HCP [0001], as long as the non-inclined vector is the same one.
(2) IL slightly scatters and does not fall into one plane as long as the constraint **P_1_** is not parallel to one of the basal axes of the reference coordination system. The only exception is HCP-to-BCC with **P_1_**//$\left[2\bar{11}0\right]$, which restricts all IL points into one plane.
(3) LPR zones I and II share the same plane containing all rotation axes and the same zone axes covering all HP.
(4) For BCC-to-HCP athermal case, the rotation axis is always parallel to [110], i.e., slip plane (110) normal, which will turn into (0001) of the HCP precipitate. All HP normal and IL belong to the (110) plane. This feature repeats in HCP-to-BCC athermal transformation.

## 7. Discussion

### 7.1. Equivalence of Phase Transformation Direction

It is often assumed that the predicted crystallographic features of a phase transformation should have no difference no matter which phase is chosen as the matrix. That is to say, for the same transformation system, using a different phase as the matrix will derive the same predicted results. This work further investigated the equivalence of phase transformation direction.

Figure 8A shows the athermal precipitation of a BCC-to-HCP transformation with LPR *γ*_2_ = 1 and *γ*_1_ varying in a full range of (1 to 1.2247). Figure 8B shows that for an HCP-to-BCC transformation. It is found that they share the same LPR range, and the crystallographic features are mutually exchangeable by considering lattice conversion between BCC and HCP. The equivalence of phase transformation direction seems true for athermal precipitation. However, this is not true for isothermal precipitation. This can be confirmed by comparing the calculated results in Figure 6A and Figure 7A, both of which are controlled by a non-inclined vector parallel to the matrix’s Burgers vector. Apparently, the LPR range of BCC-to-HCP isothermal precipitation is different from that of HCC-to-HCP. The non-equivalence of phase transformation direction was also verified in the FCC–BCC system [25]. The equivalence of phase transformation direction becomes true as long as both cases are sharing the same rotation axis in the reference coordination system.

### 7.2. Blank LPR Zone

A transformation system falling into the blank LPR zone means its LPR is out of the rational range of those that can produce an invariant line. If not, it is still possible to cover it into the known LPR range by inducing coincidence site lattice (CSL) and near CSL (NCSL) to the system. This is the reason why the above systematic investigation of the LPR range is limited to the range (0, 2). For this consideration, an LPR greater than or equal to 2 will be using the CSL concept to reduce the lattice parameter until it falls back below 2. One example is the transformation α-Mg⟶*γ*-Mg_17_Al_12_. The BCC precipitation has a very large lattice parameter, and the initial LPR *γ_2_* is 4.852. This value has been replaced with a value equal to one third of the initial LPR, as shown in the first row of Table 1. This is based on a consideration that the BCC lattice parameter is around three times of that along the ‘**a**’ axis of HCP [26].

### 7.3. Competition between Isothermal and Athermal Precipitation

Revisiting Table 2, an interesting finding is that the IDE model constraint of one-step rotation of isothermal precipitation is reciprocal to that of athermal precipitation. The former requires a non-inclined direction, which is usually parallel to a Burgers vector of the matrix phase and is accompanied with an invariant normal perpendicular to this direction. The latter, however, keeps a non-inclined plane normal of a slip plane, and this produces an invariant line on it. In view of this point, the classic martensitic phase transformation as a typical athermal transformation [27] is apparently not the same as athermal precipitation. For instance, Zheng et al. found that the martensitic transformation in the Heusler alloy Ni–Mn–Sn is athermal in nature, although a time-dependent effect is observed through DSC-interrupted measurements [27]. Consequently, isothermal precipitation has a much closer relationship with athermal precipitation rather than with athermal martensitic transformation.

It is revealed that a non-inclined crystallographic feature, i.e., a Burgers vector for isothermal precipitation or a slip plane for athermal precipitation, plays an important role to define the final OR, HP, and IL of the transformation product. It provides us a clear illustration that a precipitation is dominated by some important invariant deformation elements (IDEs), such as a Burgers vector of dislocation and a slip plane of the slip system. By keeping either of these crystalline items non-inclined, precipitation can produce predictable crystallographic features including OR, HP, and IL. Standing on this point, interface structure covering dislocation structure can be derived by a notable O-lattice model [28]. With the consideration of the interface energy, a three-dimensional morphology of a precipitate can also be created by using the surface energy solution, e.g., the Wulff plot shown in [29].

### 7.4. Experimental Identification of Precipitation Type

According to the detailed full LPR range investigation of the isothermal and athermal precipitation in BCC and HCP systems (see Figure 6, Figure 7 and Figure 8), it is possible to assign a precipitation of the HCP–BCC system to either isothermal or athermal precipitation according to its habit plane Miller index. For the BCC-to-HCP transition, the observed habit plane in a zone axis of BCC <11*w*> belongs to isothermal precipitation if *w* ≠ 0, or athermal precipitation if *w* = 0. For the HCP-to-BCC transition, a habit plane in a zone axis of $\left[2\bar{11}0\right]$ can always be assigned to isothermal precipitation; otherwise, that in the [0001] zone axis can be assigned to athermal precipitation.

## 8. Conclusions

A comparative investigation of the crystallographic features of isothermal and athermal precipitation in an HCP–BCC system was carried out in a full lattice parameter ratio range containing two variants by using the IDE model for diffusional phase transformation. On the basis of the successful evaluation of the IDE model to the HCP–BCC precipitation system, the full LPR range investigation provides the following overview of the complicated two-variant precipitation system.

1. It is revealed that, whether it is isothermal or athermal precipitation, a non-inclined crystallographic feature, i.e., a Burgers vector for isothermal precipitation or a slip plane for athermal precipitation, plays an important role to define the final OR, HP, and IL of the transformation product.
2. It is found that the equivalence of phase transformation direction becomes true as long as both cases are sharing the same rotation axis in the reference coordination system. This is the case for athermal precipitation; however, it is not true for isothermal precipitation.
3. The ratio *c*/*a* of the hexagonal phase plays an important role in the determination of the general Bain strain between the BCC and HCP phases. However, its effect to the strain-induced rotation presents in isothermal precipitation but is limited in athermal precipitation.
4. According to the observed habit plane Miller index, a precipitation in the HCP–BCC system can be assigned to either isothermal precipitation if the Miller index falls into a zone axis of BCC <11w>(w ≠ 0) or HCP $\left[2\bar{11}0\right]$, or athermal precipitation when it is found to locate in a zone axis of BCC <11w] (w = 0) or HCP [0001].

## Figures and Tables

**Table 1:** Lattice parameters of phases investigated in this work.

System (1)		Phase	Structure	Lattice Parameters				Mode	Ref.
				a	c	*γ_1_*	*γ_2_*		
HCP ↓ BCC	m	*α*-Mg	HCP	*0.321*	0.518	1.095	1.614	Isothermal	[21] [22]
	p	*γ*-Mg_17_Al_12_	BCC	1.055 (2)					
	m	α-Zr	HCP	0.321	0.511	1.031	1.592	Isothermal	[6] [5]
	p	β-Nb	BCC	0.331					
BCC ↓ HCP	m	*β*-Ti	BCC	0.325		1.09	1.58	Athermal	[3] [4]
	p	*α*-Ti	HCP	0.296	0.469				
	m	*α*-Mo	BCC	0.315		1.05	1.57	Isothermal	[8] [8]
	p	Mo_2_C	HCP	0.300	0.472				
	m	*β*-Li	BCC	0.354		1.11	1.61	Athermal	[19]
	p	*α*-Mg	HCP	0.319	0.513				

(1) “m” denotes matrix and “p” denotes precipitate. (2) One third of this value (0.351) is used for calculation. Phase γ-Mg_17_Al_12_ has a large BCC unit cell that can be regarded as made up from 27 subcells stacked 3 × 3 × 3, with each subcell having a structure that is approximately BCC [15,23]. For convenience of calculation, efficient lattice parameter of γ-Mg_17_Al_12_ is taken as 1/3 of published lattice parameter, i.e., 0.355 nm.

**Table 2:** Calculation details and intermediate results of crystallographic features of the five investigated transformations.

No	System	Item	Matrix System	Reference System	Main Strain	Non-Inclined Item
1	α-Mg ↓ γ-Mg17Al12	**P_1_**	[0001]	[001]	*η_1_* = 1.090	Non-inclined direction
		**P_2_**		[0 0 0.958]	*η_2_* = 0.890	
		**Q_1_**		(0.776 0.631 0)	*η_3_* = 0.953	Invariant normal
		**Q_2_**		(0.708 0.706 0)		
		**u**/*θ*	[001]/5.77°	[001]/5.77°		
2	α-Zr ↓ β-Nb	**P_1_**	[0001]	[001]	*η_1_* = 1.031	Non-inclined direction
		**P_2_**		[0 0 0.916]	*η_2_* = 0.842	
		**Q_1_**		(0.935 0.355 0)	*η_3_* = 0.916	Invariant normal
		**Q_2_**		(0.907 0.422 0]		
		**u**/*θ*	[001]/4.15°	[001]/4.15°		
3	β-Ti ↓ α-Ti	**P_1_**		[0 0.836 1]	*η_1_* = 1.020	Invariant line
		**P_2_**		[0 0.932 0.911]	*η_2_* = 1.115	
		**Q_1_**	(1 1 0)	(1 0 0)	*η_3_* = 0.911	Non-inclined plane
		**Q_2_**		(0.980 0 0)		
		**u**/*θ*	[110]/5.78°	[100]/5.78°		
4	α-Fe ↓ Mo2C	**P_1_**	[110]	[100]	*η_1_* = 1.059	Non-inclined direction
		**P_2_**		[1.059 0 0]	*η_2_* = 1.166	
		**Q_1_**		(0 0.510 1]	*η_3_* = 0.952	Invariant normal
		**Q_2_**		(0 0.437 1.050]		
		**u**/*θ*	[110]/3.99°	[100]/3.99°		
5	β-Li ↓ α-Mg	**P_1_**		[0 0.931 1]	*η_1_* = 1.024	Invariant line
		**P_2_**		[0 1.027 0.901]	*η_2_* = 1.103	
		**Q_1_**	(1 1 0)	(1 0 0)	*η_3_* = 0.901	Non-inclined plane
		**Q_2_**		(0.976 0 0)		
		**u**/*θ*	[110]/5.79°	[100]/5.79°		

**Table 3:** Comparison of crystallographic features of HCP–BCC diffusional transformations between literature and current work.

System	Item	Experimental	Current Work		Other Work		
			Calculated	Diff.	Calculated	Diff.	Ref.
α-Mg ↓ γ-Mg17Al12	OR	(0001)//(110)	(0001)//(110)	0°	(0001)//(110)	0°	[21,22]
		[11–20] 0.3°⟶[1-1-1]	[11–20] 0.6°⟶[1-1-1]	0.3°	[11–20] 0.53°⟶[1-1-1]	0.23°	
		[1–100] 0.3°⟶[1-12]	[1–100] 0.6°⟶[1-12]	0.3°	[1–100] 0.53°⟶[1-12]	0.23°	
	HP	(0001)/(110)	(0001)	0°	(0001)	0°	
	IL	[7, 2, −90]	[19, 7, −26, 0]/[7, −7, 0]	2.72°	[4.2622, 1, −5.2622, 0]	1.9°	
α-Zr ↓ β-Nb	OR	(0001)//(110)	(0001)//(110)	0°	(0001)//(110)	0°	[5,6]
		[11–20] 1.5°⟶[1-1-1]	[11–20] 0.9°⟶[1-1-1]	0.2°	[11–20] 1.1°⟶[1-1-1]	0.2°	
		[1–100] 1.5°⟶[1-12]	[1–100] 0.9°⟶[1-12]	0.2°	[1–100] 1.1°⟶[1-12]	0.2°	
	HP	(0001)/(110)	(0001)/(110)	0°	(0001)/(110)	0°	
	IL	[7.09, −1.09, −6, 0]	[7, −1, −6, 0]/[5, −5, 17]	2.56°	[5.3398, −1, −4.3398, 0]	1.93°	
β-Ti ↓ α-Ti	OR	(0001) 0.6°⟶(110)	(0001)//(110)	0.6°	(0001)//(110)	0.6°	[24]
		[11–20] 0.7°⟶([1-1-1]	[11–20] 0.5°⟶[1-1-1]	0.2°	[11–20] 0.5°⟶[1-1-1]	0.2°	
		[1–100] 0.7°⟶([1-12]	[1–100] 0.5°⟶[1-12]	0.2°	[1–100] 0.5°⟶[1-12]	0.2°	
	HP	(−3.6 4.6 −1 −0.097)/(−13, 10.2, 10.1)	(−0.64, 0.543, 0.543)	2.37°	(−13, 11.5, 10.3)	2.99°	
	IL	[3.2, 1, −4.2, 0]/[5, 2.6, 2.8]	[0.767, 0.453, 0.453]	2.81°	[5, 2.8, 3.2]	3.171°	
α-Mo ↓ Mo2C	OR	(0001)//(110)	(0001)//(110)	0°	(0001)//(110)	0°	[7,8]
		[11–20]⟶[1-1-1]	[11–20] 1.2°⟶[1-1-1]	0.2°	[11–20] 0.36°⟶[1-1-1]	0.3°	
		[1–100]⟶[1-12]	[1–100] 1.2°⟶[1-12]	0.2°	[1–100] 0.3°⟶[1-12]	0.3°	
	HP	(11–23)/(−301) *	(−2.772, 0, 1)	2.32°	(0.90, 0, 0.304)	0.23°	
	IL	[1–100]/[113]*	[1, 1, 2.772]	1.79°	[0.304,0.304,0.9]	0.29°	
β-Li ↓ α-Mg	OR	(0001)//(110)	(0001)//(110)	0°	(0001)//(110)	0°	[20]
		[11–20] 0.5°⟶[1-1-1]	[11–20] 0.6°⟶[1-1-1]	0.6°	[11–20] 0.524°⟶[1-1-1]	0.6°	
		[1–100] 0.5°⟶[1-12]	[1–100] 0.6°⟶[1-12]	0.6°	[1–100] 0.524°⟶[1-12]	0.6°	
	HP	{3–410}/(−9, 9, 11) *	(0.532, 0.532, 0.659)	0.38°	Not reported	--	
	IL	[52 –70]/[11, −11, 18] *	[0.482, −0.482, 0.732]	2.13°	Not reported	--	

* Not reported. This is derived by the current author by revisiting the published paper.

## Data Availability

The study did not report any data.
